# A Higher-Order Ising Model with Gradient-Free Update

**DOI:** 10.3390/axioms14120879

**Published:** 2025-11-28

**Authors:** Gengsheng L. Zeng

**Affiliations:** Department of Computer Science, Utah Valley University, Orem, UT 84058, USA

**Keywords:** Ising model, optimization, machine learning, neural network, inverse functions, logic circuits, exclusive-OR gate, decoding of error-correcting codes, 68T01, 68T05, 68T20, 68W01, 68W40, 90B80, 90C09, 90C56, 94C11, 94D10, 97P80

## Abstract

The Ising model is able to memorize some patterns or solutions as stable states. An Ising network may automatically converge to a pre-stored solution for a random input. However, in many cases, the Ising model cannot perform this task. The gap is that for a set of desired patterns, one may not be able to construct an Ising model such that the desired patterns are the stable solutions of the Ising model. The Ising model has limited power, because its energy function is limited to a second-order polynomial. Our research outline is as follows. This paper extends the conventional Ising model so that it has wider applications, where the Hebbian rule no longer works. The extended model does not have a limit on the order of the energy function. The extended Ising is defined by combining all desired patterns in a product. Our findings are that the extended Ising model has explicit closed-from update formulas, which do not require the evaluation of gradients. Thus, no network training is necessary. The update algorithm takes finite steps to reach a local minimum.

## 1. Introduction

The classical Ising model is expressed as a quadratic function [1–5]. This quadratic function is commonly referred to as a Hamiltonian, for example [3,5],

(1) $$E=-\sum_{i\neq j}\sum_{j}w_{ij}x_{i}x_{j}-\sum_{i}b_{i}x_{i}=-\frac{1}{2}xWx^{T}-xB^{T},$$

where x is a row vector and each element in $x,x_{i}$ is a binary variable, taking values either in {−1, 1} or in {0, 1}. The term ‘Hamiltonian’ is commonly used in physics. This term is also known as the ‘energy function’ in physics, the ‘objective function’ in mathematics and optimization, and the ‘cost function’ or ‘loss function’ in neural network training.

In usual machine learning, the cost function E is used to iteratively determine (that is, to train) the network parameters W and B. However, in the context of the Ising model, both W and B are calculated or designed. Once the Ising model is established for a particular application, a random input is fed to the Ising model. An iterative optimization algorithm is then used to minimize the energy function (1) to reach a desired pattern x. A popular optimization algorithm is the stochastic Boltzmann algorithm, which has a fast Field Programmable Gate Array (FPGA) implementation [5–7].

The stochastic Boltzmann algorithm is essentially a stochastic gradient descent algorithm; it does not always move in the downhill direction [8–12]. It moves in the uphill direction with some small probability, hoping to find a global solution. In this paper, we propose a deterministic algorithm without using the gradient.

In a classic Ising model (1), the diagonal elements of the symmetric matrix W are all zeros, and B is a row vector. Obtaining W and B for a given problem is not an easy task, because we may not have an Ising model for the problem at hand. Now let us give a brief introduction to how the symmetric matric W is obtained using Hebb’s rule as follows [13–16].

This paper focuses on the special case of the Ising model when the ‘temperature’ is zero. The ‘temperature’ is an indicator of the randomness or noise in the system. In this special case, the Ising model is a Hopfield network [17–20].

The Hebbian rule in a Hopfield network is the principle that if two neurons are simultaneously active (or both inactive), their connection strengthens. Conversely, if their activations are different, their connection weakens [14]. To express this principle mathematically, the Hebbian rule gives a closed-form formula to obtain W [21,22] as follows. If the Ising model has M stable states: $x^{S_{1}},x^{S_{2}},\ldots ,x^{S_{M}},w_{ij}$ is calculated as

(2) $$w_{ij}=x_{i}^{s_{1}}x_{j}^{s_{1}}+x_{i}^{s_{2}}x_{j}^{s_{2}}+\cdots +x_{i}^{s_{M}}x_{j}^{s_{M}},$$

(3) $$w_{ii}=0.$$

There is no known general formula to find B, which, in general, is not unique for a problem.

In the following, we are showing two small examples of how to use an Ising model to find desired solutions. In the following, we are showing two small examples of how to use an Ising model 71 to find desired solutions.

### 1.1. Example 1: A Logic OR Gate

The Ising model has applications in logic circuits [23]. The following is an example of an Ising model that remembers the logic OR gate function (see Table 1) [24]. Each row in Table 1 is a ‘solution’, which the Ising model is supposed to remember. Using standard Ising model notation, we use {−1, 1} instead of {0, 1} to represent a binary number. Each ‘solution’ is a ‘stable state’ of the Hamiltonian; it is a local minimum of the energy function. In an Ising model, each variable $x_{i}$ is a unit or a neuron.

According to Hebbian rule, the matrix W is calculated as the average of the ‘outer products’ $x^{T}x$, where x is a stable state. Here, x is a row vector; $x^{T}$ is its transpose. The outer product $x^{T}x$ is a matrix. In the case of the logic OR function in Table 1, there are four stable states. Each row in Table 1 is an instance of the row vector x. Then we set the diagonal elements of W to zeros. The W matrix can be scaled by an arbitrary positive normalization factor. In other words, if E is a valid Hamiltonian (that is, an energy function), aE is also valid if a>0. A valid W for the OR gate problem is given as

(4) $$W=\left[\begin{array}{lll} 0 & 0 & 1\\ 0 & 0 & 1\\ 1 & 1 & 0 \end{array}\right].$$

By trial and error, we select

(5) $$B=\left[\begin{array}{lll} 0 & 0 & 0.5 \end{array}\right].$$

According to (1), a Hamiltonian for the OR gate problem is

(6) $$E=-\left(x_{1}x_{3}+x_{2}x_{3}\right)-0.5x_{3}.$$

By applying this Hamiltonian to all possible (random) input states x, the corresponding Hamiltonian values are listed in Table 2. The ‘stable states’ (or valid ‘solutions’) at the upper part of the table with the shaded cells have negative E values, while other unstable states at the lower part of the table have positive E values.

It is important that the value E of a stable state is lower than the E values of its neighboring unstable states. For example, {−1, −1, −1} with E=−1.5 is a stable state. Its neighbors are {−1, −1, −1} with E=0.5, {−1,1,−1} with E=0.5, and {−1,−1,1} with E=1.5.

There are many methods to minimize the Hamiltonian E. One popular method is to use a logistic function [25]

(7) $$V=\frac{1}{1+e^{-E}}.$$

to convert a continuous function into a pseudo probability distribution function on [0, 1]. After this mapping (7), one can use a probability optimization method to search for the optima of V. This conversion (7) is unnecessary if we do not use a probability optimization method, and we will not discuss this conversion any further in this paper.

We propose to use an asynchronous optimization method to update one variable at a time while fixing other variables. For example, when we consider variable $x_{i}$, we compare the values between $E\left(x_{i}=1\right)$ and $E\left(x_{i}=-1\right)$. We choose the smaller one and assign the corresponding value of $x_{i}$. The sign of $E\left(x_{i}=1\right)-E\left(x_{i}=-1\right)$ is the same as the sign of $\frac{\partial E}{\partial x_{i}}$ in the continuous variable case. Therefore, the update formula is

(8) $$x_{i}=\text{sign}\left(\sum_{j\neq i}w_{ij}x_{j}+b_{i}\right)=\text{sign}\left(-\frac{\partial E}{\partial x_{i}}\right).$$

if $\sum_{j\neq i}w_{ij}x_{j}+b_{i}=0,x_{i}$ is randomly selected to 1 or −1.

In the OR gate example, let us consider the situation of updating the variable $x_{3}$. According to the energy function (6), we have

(9) $$-\frac{\partial E}{\partial x_{3}}=x_{1}+x_{2}+0.5.$$

Table 3 shows the situations of updating $x_{3}$, while $x_{1}$ and $x_{2}$ are fixed. The last column in Table 3 shows the updated values of $x_{3}$. After one step update, the system reaches a stable state of the OR gate.

### 1.2. Example 2: A Logic XOR Gate

For some logic functions, additional binary variables are needed to form their Ising models, otherwise their Ising models do not exist [5]. This second example considers the logic exclusive-OR (XOR) gate [24], defined in Table 4 using binary values {−1, 1}.

For this example, Hebb’s rule gives

(10) $$W=\left[\begin{array}{lll} 0 & 0 & 0\\ 0 & 0 & 0\\ 0 & 0 & 0 \end{array}\right].$$

A zero matrix W indicates that this Ising model is invalid, because the update formula would be a constant for every input and there would be only one stable state. This 3-variable Ising model is inadequate to model the XOR function. One workaround solution is to introduce some auxiliary variables.

In fact, there are many problems that a direct application of the Ising model does not work. Even for the cases where the Ising model works, its Hamiltonian values for the stable states are different. See Table 2 of our OR gate example, where the Hamiltonian values for the stable states are −1.5, −0.5, −0.5 and −2.5, respectively. This is not an ideal situation. The stability for E=−0.5 and for E=−0.25 may be different. The goal of this paper is to overcome these shortcomings.

This introductory section explains what an Ising model is with two examples. In the first example, we successfully created an Ising model, and the model could be used to find the patterns satisfying the OR gate relationship. Unfortunately, the Hebbian rule failed to construct an Ising model to search for patterns that met the XOR gate relationship.

There were attempts to extend the Ising model to use higher-order terms [26–28]. In the classic Ising model (1), the terms in the energy function can only contain one or two variables. The energy function in a high-order Ising model allows terms containing three or more variables. This increase in the complexity in the energy function makes the optimization procedure more intractable because it is an NP-complete problem in general. To date there is no effective and efficient algorithm to optimize a high-order Ising energy function.

The rest of the paper is organized as follows: Section 2 will propose an extended Ising model that can solve the XOR gate problem. Section 3 will show how the new model searches for the desired patterns using the same two examples. Section 4 will discuss the architecture of the new model with primary and secondary neurons. Section 5 presents an application of the proposed method to a practical problem, decoding of an error-correcting code. Section 6 will discuss the computational complexity of the proposed update algorithm. Section 7 will summarize the innovation, impact, and potential applications of the proposed model. In Appendix A, closed-form update formulas are derived.

## 2. New Energy Function

The method we are presenting in this section is inspired by the Ising model and it overcomes the two drawbacks of the Ising model. The two drawbacks are as follows.

1. When given a set of stable states of n binary variables, it may not be possible to construct an associated Ising model with n variables, as illustrated by Example 2 (the XOR function) in the previous section. This is because the energy function of an Ising model is restricted to a quadratic form.
2. When a valid Ising model is found for a set of stable states, as illustrated by Example 1 (the OR function) in the previous section, the energy function values corresponding to those stable states may be different. Some of them are local minima.

When we are given a set of M stable states: $x^{S_{1}},x^{S_{2}},\ldots ,x^{S_{M}}$, we define the energy function E as

(11) $$E(x)=\prod_{i=1}^{M}{\left\|x-x^{S_{i}}\right\|}^{2}=\prod_{i=1}^{M}\sum_{j=1}^{n}{\left(x_{j}-x_{j}^{S_{i}}\right)}^{2},$$

which has a distinguishing property that E(x) always satisfies

(12) E(x)≥0,

and E(x)=0 if and only if x is a stable state. In other words, all stable states reach the global minimum of the energy function E(x). The M stable states $x^{S_{1}},x^{S_{2}},\ldots ,x^{S_{M}}$, are the M desired patterns, represented in {0, 1} in the rest of this paper. In fact, the variables in (11) can be represented in any two symbols for binary applications. Note that in the previous section the variables are represented in {−1, 1}.

Since the variables $x_{i}$ are in {0, 1}, we have $x_{i}^{j}=x_{i}$ for any integer j≥1. Therefore, (11) has a general form of

(13) $$E\left(x\right)=\sum_{k=0}^{2^{n}-1}a_{k}\prod_{i=1}^{n}x_{i}^{k_{i}}=\sum_{k=0}^{2^{n}-1}a_{k}x_{1}^{k_{1}}x_{2}^{k_{2}}\ldots x_{n}^{k_{n}},$$

with $k_{i}\in \{0,1\}$. We will apply this new model (11) to the two examples in the previous section.

We now provide some intuition about the extended Ising model (11). The original Ising model (1) is defined by a quadratic energy function (1). This quadratic function can have any real values including negative values. On the other hand, the extended model (11) or (13) is defined by an nth order polynomial as the energy function, where n is the number of the primary binary variables involved in the system. In the two examples presented in the previous section, n=3. In order for the Ising model to ‘store’ all the desired patterns, the desired patterns must be local minima of the energy function E. If we cannot find a second-order polynomial E in which the desired patterns are the local minima, the Ising model fails. By increasing the order of the polynomial E, we have a better chance to find such a polynomial. If we construct a polynomial E as in (11), all the desired patterns $x^{s_{i}}$ are global minima with minimal value of E=0. All other undesired patterns have energy E>0.

The energy function (11) is a polynomial of order n, which is generally greater than 2. In the following two examples, we demonstrate how our extended model works.

### 2.1. Example 1: A Logic OR Gate

The four stable states (i.e., four solutions) in this OR gate example are the four rows in Table 5 [24]. The variables are in {0, 1}.

The new energy function (11) for this example is given as

(14) $$E(x)=\left[x_{1}^{2}+x_{2}^{2}+x_{3}^{2}\right]\;\times \left[x_{1}^{2}+{\left(1-x_{2}\right)}^{2}+{\left(1-x_{3}\right)}^{2}\right]\;\times \left[{\left(1-x_{1}\right)}^{2}+x_{2}^{2}+{\left(1-x_{3}\right)}^{2}\right]\;\times \left[{\left(1-x_{1}\right)}^{2}+{\left(1-x_{2}\right)}^{2}+{\left(1-x_{3}\right)}^{2}\right]\;=\left[x_{1}+x_{2}+x_{3}\right]\;\times \left[x_{1}+1-x_{2}+1-x_{3}\right]\;\times \left[1-x_{1}+x_{2}+1-x_{3}\right]\;\times \left[1-x_{1}+1-x_{2}+1-x_{3}\right]\;=6x_{1}+6x_{2}+2x_{3}-4x_{1}x_{2}-8x_{1}x_{3}-8x_{2}x_{3}+6x_{1}x_{2}x_{3},$$

which is not a traditional Ising model because of the extra term $6x_{1}x_{2}x_{3}$. The energy function values for all inputs are listed in Table 6. All stable states have E values of zero. All non-stable states have positive E values of 2, 6, or 8.

### 2.2. Example 2: A Logic XOR Gate.

This second example concerns the logic exclusive-OR gate [24], defined in Table 7 using binary values {0, 1}.

The proposed energy function (11) for the second example is given as

(15) $$E(x)=\left[x_{1}^{2}+x_{2}^{2}+x_{3}^{2}\right]\;\times \left[x_{1}^{2}+{\left(1-x_{2}\right)}^{2}+{\left(1-x_{3}\right)}^{2}\right]\;\times \left[{\left(1-x_{1}\right)}^{2}+x_{2}^{2}+{\left(1-x_{3}\right)}^{2}\right]\;\times \left[{\left(1-x_{1}\right)}^{2}+{\left(1-x_{2}\right)}^{2}+x_{3}^{2}\right]\;=\left[x_{1}+x_{2}+x_{3}\right]\;\times \left[x_{1}+1-x_{2}+1-x_{3}\right]\;\times \left[1-x_{1}+x_{2}+1-x_{3}\right]\;\times \left[1-x_{1}+1-x_{2}+x_{3}\right]\;=3x_{1}+3x_{2}+3x_{3}-6x_{1}x_{2}-6x_{1}x_{3}-6x_{2}x_{3}+12x_{1}x_{2}x_{3},$$

which is not an Ising model because of the extra term $12x_{1}x_{2}x_{3}$. We recall from the previous section that the 3-variable Ising model does not exist for the XOR gate function. The extended Ising model exists for three variables. The energy function values for all inputs are listed in Table 8. All stable states have E values of zero. All non-stable states have positive E values of 3.

## 3. New Update Formula

In this section, we develop an asynchronous algorithm to minimize the extended Ising model’s energy function. In an asynchronous algorithm, the variables are updated in the manner of one variable at a time. We assume that we want to update the variable $x_{i}$. Let $E_{i}^{0}$ be E(x) with x being the current state except that the ith component $x_{i}$ is clamped to 0. Let $E_{i}^{1}$ be E(x) with x being the current state except that $x_{i}$ is clamped to 1. The superscript indicates clamped value; the subscript is the component index.

The proposed algorithm uses the following principle: if $E_{i}^{0}<E_{i}^{1},x_{i}$ is updated to 0. If $E_{i}^{0}>E_{i}^{1},x_{i}$ is updated to 1. If $E_{i}^{0}=E_{i}^{1},x_{i}$ is not updated (or updated to 1 or 0 randomly). In other words, the update formula is

(16) $$x_{i}=\text{step}\left(E_{i}^{0}-E_{i}^{1}\right),$$

where the step function is defined as

(17) $$\text{step}(t)=\left\{\begin{array}{l} 1,t>0\\ 0,t<0. \end{array}\right.$$

Next, we present the update formulas according to the general formula (16) for the two examples discussed in this paper.

### 3.1. Example 1: A Logic OR Gate

For the first example, the update formulas are

(18) $$x_{1}=\text{step}\left(E_{1}^{0}-E_{1}^{1}\right)\;=\text{step}\left(\left[6x_{2}+2x_{3}-8x_{2}x_{3}\right]-\left[6+6x_{2}+2x_{3}-4x_{2}-8x_{3}-8x_{2}x_{3}+6x_{2}x_{3}\right]\right)\;=\text{step}\left(-6+4x_{2}+8x_{3}-6x_{2}x_{3}\right);$$

(19) $$x_{2}=\text{step}\left(E_{2}^{0}-E_{2}^{1}\right)\;=\text{step}\left(\left[6x_{1}+2x_{3}-8x_{1}x_{3}\right]-\left[6x_{1}+6+2x_{3}-4x_{1}-8x_{1}x_{3}-8x_{3}+6x_{1}x_{3}\right]\right)\;=\text{step}\left(-6+4x_{1}+8x_{3}-6x_{1}x_{3}\right);$$

(20) $$x_{3}=\text{step}\left(E_{3}^{0}-E_{3}^{1}\right)\;=\text{step}\left(\left[6x_{1}+6x_{2}-4x_{1}x_{2}\right]-\left[6x_{1}+6x_{2}+2-4x_{1}x_{2}-8x_{1}-8x_{2}+6x_{1}x_{2}\right]\right)\;=\text{step}\left(-2+8x_{1}+8x_{2}-6x_{1}x_{2}\right).$$

### 3.2. Example 2: A Logic XOR Gate

For the second example, the update formulas are

(21) $$x_{1}=\text{step}\left(E_{1}^{0}-E_{1}^{1}\right)\;=\text{step}\left(\left[3x_{2}+3x_{3}-6x_{2}x_{3}\right]-\left[3+3x_{2}+3x_{3}-6x_{2}-6x_{3}-6x_{2}x_{3}+12x_{2}x_{3}\right]\right)\;=\text{step}\left(-3+6x_{2}+6x_{3}-12x_{2}x_{3}\right);$$

(22) $$x_{2}=\text{step}\left(E_{2}^{0}-E_{2}^{1}\right)\;=\text{step}\left(\left[3x_{1}+3x_{3}-6x_{1}x_{3}\right]-\left[3x_{1}+3+3x_{3}-6x_{1}-6x_{1}x_{3}-6x_{3}+12x_{1}x_{3}\right]\right)\;=\text{step}\left(-3+6x_{1}+6x_{3}-12x_{1}x_{3}\right);$$

(23) $$x_{3}=\text{step}\left(E_{3}^{0}-E_{3}^{1}\right)\;=\text{step}\left(\left[3x_{1}+3x_{2}-6x_{1}x_{2}\right]-\left[3x_{1}+3x_{2}+3-6x_{1}x_{2}-6x_{1}-6x_{2}+12x_{1}x_{2}\right]\right)\;=\text{step}\left(-3+6x_{1}+6x_{2}-12x_{1}x_{2}\right).$$

## 4. New Neural Network Architecture

For the new energy function (11), the proposed neural network consists of n primary neurons: $x_{1},x_{2},\ldots ,x_{n}$, and some necessary secondary neurons, such as $x_{1}x_{2},x_{1}x_{3},x_{2}x_{3},\ldots ,x_{1}x_{2}x_{3}\ldots x_{n}$. The secondary neurons are functions of the primary variables; we call them ‘helping functions’ in this paper. All these primary and secondary neurons are the terms that appear in the update formulas. We will use our two examples to illustrate the architecture of the extended model.

### 4.1. Example 1: A Logic OR Gate

**Figure 1.**
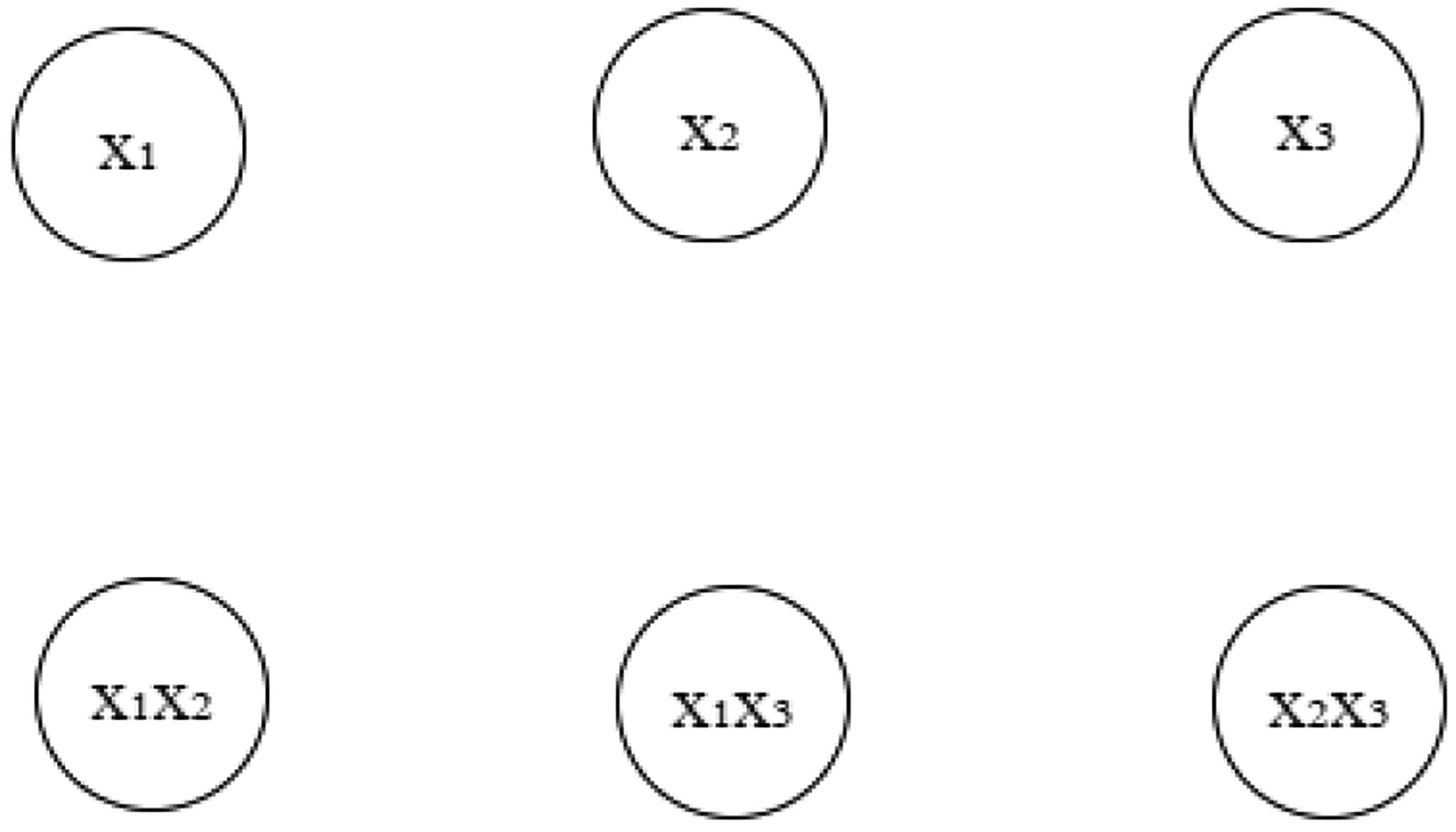
The proposed neural network consists of the primary neurons and secondary neurons. The architecture figures show the primary and secondary neurons. The primary neurons are physically stored in the computer, while the secondary neurons are computed on the flight and are not physically stored. No directional connections are shown in figures because the update procedure is described in the update formulas.

In the first example, the update formulas are given in (18)–(20). In addition to the three primary variables $x_{1},x_{2}$, and $x_{3}$, three helping functions appeared in the formulas: $x_{1}x_{2},x_{1}x_{3}$, and $x_{2}x_{3}$. Therefore, the neuron network has six neurons as shown in Figure 1. The actions of the neurons are described by the update formulas.

In Figure 1, the state of the neural network is determined by the primary neurons $x_{1},x_{2},x_{3}$, and the secondary neurons $x_{1}x_{2},x_{1}x_{3},x_{2}x_{3},x_{1}x_{2}x_{3}$ can be readily calculated as the products from the primary neurons. Therefore, we only need an update formula for the primary neurons. The inputs and the outputs are the primary neurons.

### 4.2. Example 2: A Logic XOR Gate

In the second example, the update formulas are given in (21)–(23). In addition to the three primary variables $x_{1},x_{2}$, and $x_{3}$, three helping functions appeared in the formulas: $x_{1}x_{2},x_{1}x_{3}$, and $x_{2}x_{3}$. Therefore, the neuron network has six neurons also as shown in Figure 1. Examples 1 and 2 have the same neural network architecture, but their update formulas are different.

In Figure 1, we do not show any links between the neurons. A link is a graphic symbol to indicate the dependency of how a primary neuron is influenced by other neurons. Therefore, each primary neuron should have a link from all other neurons, even though the links are not shown.

**Figure 2.**
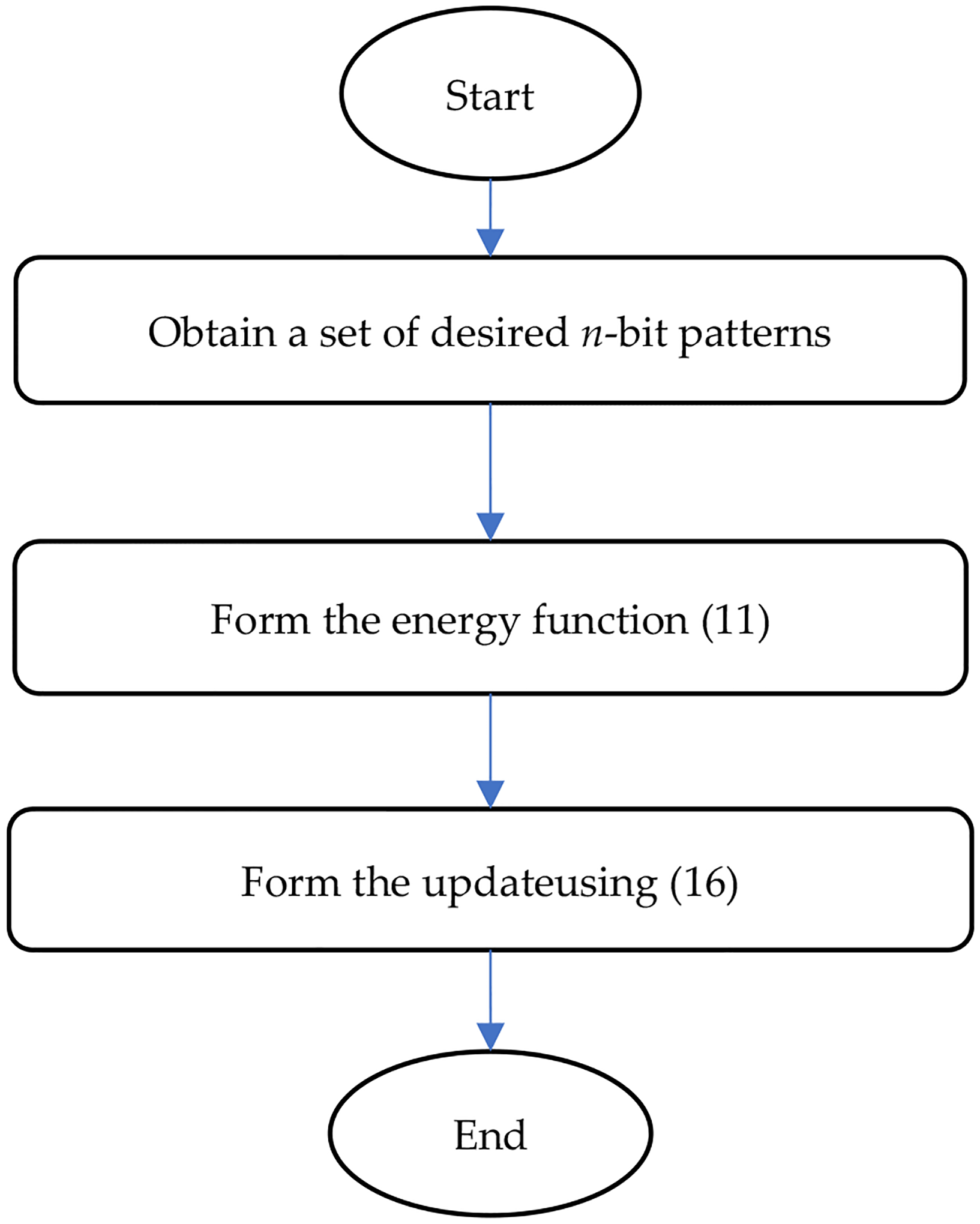
Design procedure of an extended Ising model.

**Figure 3.**
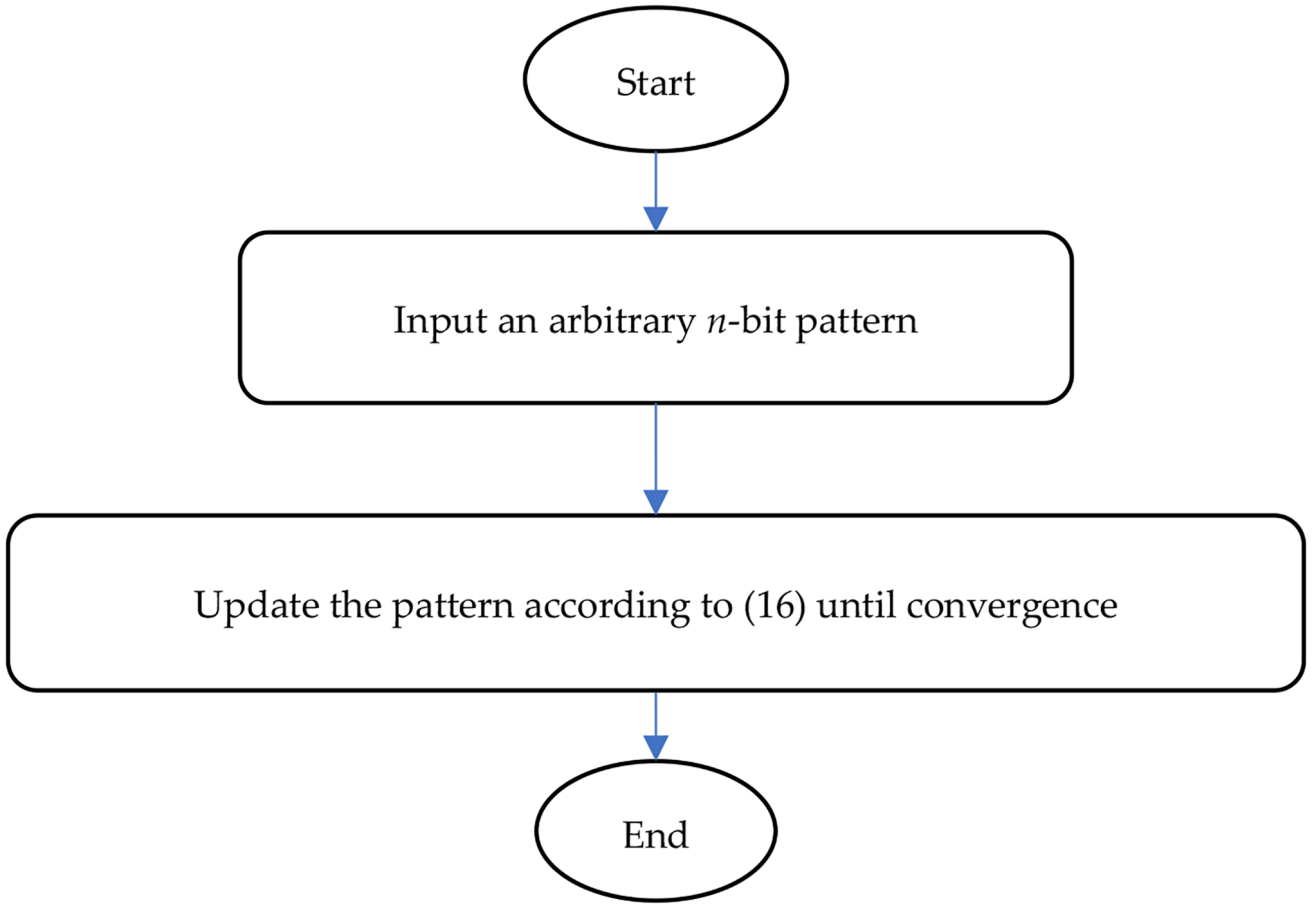
The procedure of using an extended Ising model to find the closest desired pattern.

The design procedure of an extended Ising model is summarized in a flowchart shown in Figure 2, and the flowchart for running an extended Ising model is given in Figure 3.

The experiment setup in a computer for an n-bit problem requires the storage of the n binary variables (i.e., n bits) and either the integer coefficients of the energy function or the integer coefficients of the update formulas. Each update formula contains at most $2^{n-1}$ integer coefficients, depending on the number of stable solutions in the problem. In our implementation, MATLAB^®^ R2023b was used. A practical error-correcting code with a code distance d=3 needs one step to converge. Here, one step means one iteration in an optimization algorithm, in which all variables are updated once. The computational cost is measured by the number of terms to compute in the update formulas. In our error-correcting code example, an update formula contains 21 terms, and the computation time is negligible.

## 5. A Practical Example: Error-Correcting Code Decoding

### 5.1. Principles of Error-Correcting Codes

In this section, we present a practical application of our proposed method in the field of error-correcting codes. We first use an oversimplified example to illustrate the coding/decoding problem.

In digital communications, noise introduces errors in signals. Error correction must be performed. When the sender sends a ‘0’, the receiver may receive a ‘1’ due to noise corruption. The strategy of error correction is to use data redundancy.

For example, instead of sending one bit ‘0’, one sends three bits ‘000’. Similarly, instead of sending ‘1’, one sends ‘111’. This error-correcting code has two codewords: 000 and 111. Here, the code length (i.e., the block size) is 3. At the receiving end, any non-codeword message is corrected into the closest codeword. For example, ‘101’ will be decoded as ‘111’, and ‘010’ will be decoded as ‘000’. In order to correct for t errors in a block, the code distance must be at least d=2t+1. The code distance is the minimum Hamming distance between the code words. The Hamming distance between two sequences is the number of symbols that are different between them. The Hamming distance between ‘000’ and ‘111’ is d=3.

### 5.2. Energy Function for Error-Correcting Codes

The conventional approach to verify whether a message (i.e., a binary row vector) x is a codeword is to compute the syndrome xH, where H is the parity-check matrix associated with the error-correcting code. The detection criterion is that the syndrome is a zero vector (mod 2) if and only if x is a codeword. The parity-check matrix is useful in correcting the errors. However, for a general nonlinear code, this parity-check matrix does not exist.

Let us consider an error-correcting code, which consists of four codewords as shown in Table 9. The code length is n=6; the code Hamming distance is d=3. This code is able to correct for one error and detect two errors in a block.

Using our proposed methodology, the energy function is defined as

(24) $$E(x)=\prod_{i=1}^{4}\sum_{k=1}^{6}{\left(x_{k}-c_{k}^{\left(i\right)}\right)}^{2}=\prod_{i=1}^{4}\sum_{k=1}^{6}\left[c_{k}^{\left(i\right)}+\left(1-2c_{k}^{\left(i\right)}\right)x_{\text{k}}\right].$$

Explicitly, this is a 4th-order polynomial.


(25) $$E(x)=\left(x_{1}+x_{2}+x_{3}+x_{4}+x_{5}+x_{6}\right)\;\times \left(3-x_{1}-x_{2}-x_{3}+x_{4}+x_{5}+x_{6}\right)\;\times \left(3+x_{1}+x_{2}+x_{3}-x_{4}-x_{5}-x_{6}\right)\;\times \left(6-x_{1}-x_{2}-x_{3}-x_{4}-x_{5}-x_{6}\right).$$

This energy function (25) does not require parity-check matrix. The update formulas to minimize (25) are as follows.


(26) $$x_{1}=\text{step}\left(E\left(x_{1}=0\right)-E\left(x_{1}=1\right)\right)\;=\text{step}(\left(x_{2}+x_{3}+x_{4}+x_{5}+x_{6}\right)\times \left(3-x_{2}-x_{3}+x_{4}+x_{5}+x_{6}\right)\;\times \left(3+x_{2}+x_{3}-x_{4}-x_{5}-x_{6}\right)\times \left(6-x_{2}-x_{3}-x_{4}-x_{5}-x_{6}\right)\;-\left(1+x_{2}+x_{3}+x_{4}+x_{5}+x_{6}\right)\times \left(2-x_{2}-x_{3}+x_{4}+x_{5}+x_{6}\right)\;\times \left(4+x_{2}+x_{3}-x_{4}-x_{5}-x_{6}\right)\times \left(5-x_{2}-x_{3}-x_{4}-x_{5}-x_{6}\right))\;=\text{step}(40x_{2}+40x_{3}+8x_{4}+8x_{5}+8x_{6}-8x_{2}x_{4}-8x_{2}x_{5}-8x_{2}x_{6}-8x_{3}x_{4}-8x_{3}x_{5}\;-8x_{3}x_{6}-8x_{4}x_{5}-8x_{4}x_{6}-8x_{5}x_{6}+8x_{2}x_{4}x_{5}+8x_{2}x_{4}x_{6}+8x_{2}x_{5}x_{6}+8x_{3}x_{4}x_{5}+8x_{3}x_{4}x_{6}\;+8x_{3}x_{5}x_{6}-40),$$

(27) $$x_{2}=\text{step}\left(E\left(x_{2}=0\right)-E\left(x_{2}=1\right)\right)\;=\text{step}\left(\left(x_{1}+x_{3}+x_{4}+x_{5}+x_{6}\right)\times \left(3-x_{1}-x_{3}+x_{4}+x_{5}+x_{6}\right)\right.\;\times \left(3+x_{1}+x_{3}-x_{4}-x_{5}-x_{6}\right)\times \left(6-x_{1}-x_{3}-x_{4}-x_{5}-x_{6}\right)\;-\left(1+x_{1}+x_{3}+x_{4}+x_{5}+x_{6}\right)\times \left(2-x_{1}-x_{3}+x_{4}+x_{5}+x_{6}\right)\;\left.\times \left(4+x_{1}+x_{3}-x_{4}-x_{5}-x_{6}\right)\times \left(5-x_{1}-x_{3}-x_{4}-x_{5}-x_{6}\right)\right)\;=\text{step}\left(40x_{1}+40x_{3}+8x_{4}+8x_{5}+8x_{6}-8x_{1}x_{4}-8x_{1}x_{5}-8x_{1}x_{6}-8x_{3}x_{4}-8x_{3}x_{5}-8x_{3}x_{6}\right.\;-8x_{4}x_{5}-8x_{4}x_{6}-8x_{5}x_{6}+8x_{1}x_{4}x_{5}+8x_{1}x_{4}x_{6}+8x_{1}x_{5}x_{6}+8x_{3}x_{4}x_{5}+8x_{3}x_{4}x_{6}+8x_{3}x_{5}x_{6}\;\left.-40\right),$$

(28) $$x_{3}=\text{step}\left(E\left(x_{3}=0\right)-E\left(x_{3}=1\right)\right)\;=\text{step}\left(\left(x_{1}+x_{2}+x_{4}+x_{5}+x_{6}\right)\times \left(3-x_{1}-x_{2}+x_{4}+x_{5}+x_{6}\right)\right.\;\times \left(3+x_{1}+x_{2}-x_{4}-x_{5}-x_{6}\right)\times \left(6-x_{1}-x_{2}-x_{4}-x_{5}-x_{6}\right)\;-\left(1+x_{1}+x_{2}+x_{4}+x_{5}+x_{6}\right)\times \left(2-x_{1}-x_{2}+x_{4}+x_{5}+x_{6}\right)\;\left.\times \left(4+x_{1}+x_{2}-x_{4}-x_{5}-x_{6}\right)\times \left(5-x_{1}-x_{2}-x_{4}-x_{5}-x_{6}\right)\right)\;=\text{step}\left(40x_{1}+40x_{2}+8x_{4}+8x_{5}+8x_{6}-8x_{1}x_{4}-8x_{1}x_{5}-8x_{1}x_{6}-8x_{2}x_{4}-8x_{2}x_{5}-8x_{2}x_{6}\right.\;-8x_{4}x_{5}-8x_{4}x_{6}-8x_{5}x_{6}+8x_{1}x_{4}x_{5}+8x_{1}x_{4}x_{6}+8x_{1}x_{5}x_{6}+8x_{2}x_{4}x_{5}+8x_{2}x_{4}x_{6}+8x_{2}x_{5}x_{6}\;-40),$$

(29) $$x_{4}=\text{step}\left(E\left(x_{4}=0\right)-E\left(x_{4}=1\right)\right)\;=\text{step}\left(\left(x_{1}+x_{2}+x_{3}+x_{5}+x_{6}\right)\times \left(3-x_{1}-x_{2}-x_{3}+x_{5}+x_{6}\right)\right.\;\times \left(3+x_{1}+x_{2}+x_{3}-x_{5}-x_{6}\right)\times \left(6-x_{1}-x_{2}-x_{3}-x_{5}-x_{6}\right)\;-\left(1+x_{1}+x_{2}+x_{3}+x_{5}+x_{6}\right)\times \left(4-x_{1}-x_{2}+x_{3}+x_{5}+x_{6}\right)\;\left.\times \left(2+x_{1}+x_{2}+x_{3}-x_{5}-x_{6}\right)\times \left(5-x_{1}-x_{2}-x_{3}-x_{5}-x_{6}\right)\right)\;=\text{step}\left(8x_{1}+8x_{2}+8x_{3}+40x_{5}+40x_{6}-8x_{1}x_{2}-8x_{1}x_{3}-8x_{2}x_{3}-8x_{1}x_{5}-8x_{1}x_{6}-8x_{2}x_{5}\right.\;-8x_{2}x_{6}-8x_{3}x_{5}-8x_{3}x_{6}+8x_{1}x_{2}x_{5}+8x_{1}x_{2}x_{6}+8x_{1}x_{3}x_{5}+8x_{1}x_{3}x_{6}+8x_{2}x_{3}x_{5}+8x_{2}x_{3}x_{6}\;\left.-40\right),$$

(30) $$x_{5}=\text{step}\left(E\left(x_{5}=0\right)-E\left(x_{5}=1\right)\right)\;=\text{step}\left(\left(x_{1}+x_{2}+x_{3}+x_{4}+x_{6}\right)\times \left(3-x_{1}-x_{2}-x_{3}+x_{4}+x_{6}\right)\right.\;\times \left(3+x_{1}+x_{2}+x_{3}-x_{4}-x_{6}\right)\times \left(6-x_{1}-x_{2}-x_{3}-x_{4}-x_{6}\right)\;-\left(1+x_{1}+x_{2}+x_{3}+x_{4}+x_{6}\right)\times \left(4-x_{1}-x_{2}-x_{3}+x_{4}+x_{6}\right)\;\left.\times \left(2+x_{1}+x_{2}+x_{3}-x_{4}-x_{6}\right)\times \left(5-x_{1}-x_{2}-x_{3}-x_{4}-x_{6}\right)\right),\;=\text{step}\left(8x_{1}+8x_{2}+8x_{3}+40x_{4}+40x_{6}-8x_{1}x_{2}-8x_{1}x_{3}-8x_{2}x_{3}-8x_{1}x_{4}-8x_{1}x_{6}-8x_{2}x_{4}\right.\;-8x_{2}x_{6}-8x_{3}x_{4}-8x_{3}x_{6}+8x_{1}x_{2}x_{4}+8x_{1}x_{2}x_{6}+8x_{1}x_{3}x_{4}+8x_{1}x_{3}x_{6}+8x_{2}x_{3}x_{4}+8x_{2}x_{3}x_{6}\;\left.-40\right),$$

(31) $$x_{6}=\text{step}\left(E\left(x_{6}=0\right)-E\left(x_{6}=1\right)\right)\;=\text{step}\left(\left(x_{1}+x_{2}+x_{3}+x_{4}+x_{5}\right)\times \left(3-x_{1}-x_{2}-x_{3}+x_{4}+x_{5}\right)\right.\;\times \left(3+x_{1}+x_{2}+x_{3}-x_{4}-x_{5}\right)\times \left(6-x_{1}-x_{2}-x_{3}-x_{4}-x_{5}\right)\;-\left(1+x_{1}+x_{2}+x_{3}+x_{4}+x_{5}\right)\times \left(4-x_{1}-x_{2}+x_{3}+x_{4}+x_{5}\right)\;\left.\times \left(2+x_{1}+x_{2}+x_{3}-x_{4}-x_{5}\right)\times \left(5-x_{1}-x_{2}-x_{3}-x_{4}-x_{5}\right)\right)\;=\text{step}\left(8x_{1}+8x_{2}+8x_{3}+40x_{4}+40x_{5}-8x_{1}x_{2}-8x_{1}x_{3}-8x_{2}x_{3}-8x_{1}x_{4}-8x_{1}x_{5}-8x_{2}x_{4}\right.\;-8x_{2}x_{5}-8x_{3}x_{4}-8x_{3}x_{5}+8x_{1}x_{2}x_{4}+8x_{1}x_{2}x_{5}+8x_{1}x_{3}x_{4}+8x_{1}x_{3}x_{5}+8x_{2}x_{3}x_{4}+8x_{2}x_{3}x_{5}\;\left.-40\right).$$

## 6. Convergence and Complexity

In an n-bit problem, there are n primary neurons and there are at most $2^{n}-n$ secondary neurons. The n primary neurons are the inputs and the outputs as well. There are n update rules (i.e., formulas) for the primary neurons.

The proposed network has explicit closed-form update formulas; thus, no training is required. The network is able to memorize a set of solutions (i.e., stable states). When a random n-bit input is assigned to the primary neurons, the network will update the neurons using the update rules. Each rule has a nonlinear activation function: step (t) as defined in (17). We assume that one neuron is updated at a time. We also assume that when t=0, the function step (t) does not activate any updates for the variable bit in concern. When no action is performed for one particular neuron (i.e., a particular bit), a different neuron (i.e., a different bit) will be selected for the potential update. The update actions are summarized for our three examples as follows.

### 6.1. Example 1: A Logic OR Gate

In Table 10, all possible inputs are considered. If the initial condition is a stable state, the update formulas do not activate anything. When the initial condition is not a stable state, each update is guaranteed to reduce the energy function E(x). At most two iterations are required to converge to a stable state. In Table 10, different update rules lead to different results in Steps 1 and 2.

### 6.2. Example 2: A Logic XOR Gate

In Table 11, all possible inputs are considered. If the initial condition is a stable state, the update formulas do not activate anything. When the initial condition is not a stable state, each update is guaranteed to reduce the energy function E(x). At most, one step is required to converge to a stable state. In Table 10, different update rules lead to different results in Step 1.

In general, the computing complexity analysis for the proposed optimization algorithm can be performed using the closed-form expression (A8) in Appendix A. For an n-bit Ising model, there are n primary neurons; there are no secondary neurons. Each update thus has the computational complexity of O(n). For an extended Ising model with the number of the secondary neurons being the same order of n, the computational complexity is still O(n). In the worst case, the total number of primary and secondary neurons is $2^{n}$, and the worst-case computational complexity for each update is $O\left(2^{n-1}\right)$, which is not practical for a large n. Therefore, the number of secondary neurons in an extended model plays an important role in computational complexity analysis.

### 6.3. Example 3: Error-Correcting Code Decoding

Here is a practical example in error-correcting decoding as presented in Section 5, where the code length is 6 and code distance is 3. In Table 12, all possible inputs are considered. If the initial condition is a codeword, the update formulas do not activate anything. When the initial condition is not a codeword, each update is guaranteed to reduce the energy function E(x). At most, two steps are required to converge to a codeword.

For a general error-correcting code with a code Hamming distance d, the proposed decoding algorithm takes at most (d−1)/2 steps to converge to its closest codeword.

Unlike the popular nondeterministic Boltzmann update algorithm, our proposed algorithm is deterministic and is guaranteed to monotonically minimize the energy function, reaching a local minimum. In our applications, the goal is to find a local minimum.

For binary applications, the energy function and its update procedure only use integer calculations; the computation accuracy is exact with no errors. In most cases, our proposed method converges with the exact solution in one step. Therefore, convergence curves are unnecessary. Using (13), the runtime is O(tnm), where t is the number of iterations needed to converge, and m is number of terms in the energy function. Using (11), the runtime is O(tnM), where M is the number of stable states.

#### 6.3.1. Example 1: The OR-Gate Logic Circuit

In this easiest case, the classical Ising model has a standard quadratic energy function and the extended Ising model has a fourth-order energy function. The Ising model uses the stochastic Boltzmann algorithm; the number of steps to converge is a random number. On the other hand, the extended Ising model uses a deterministic algorithm which takes at most two steps to converge, as shown in Table 12. The deterministic algorithm, in general, has shorter convergent times.

#### 6.3.2. Example 2: The XOR-Gate Logic Circuit

The classical Ising model is unable to model the XOR gate.

A higher-order Ising model [26] with an energy function of $E=\frac{1+x_{1}x_{2}x_{3}}{2}$. The main difference between the optimization method in [26] and our optimization method is as follows. The update method in [26] is the traditional gradient descent algorithm with annealing coefficients and oscillators, while our method does not use the gradients. The annealing method is a stochastic algorithm and takes a longer time to converge. On the other hand, our update formulas are deterministic. As shown in Table 11, it takes only one step for our algorithm to converge to a correct solution with an arbitrary input.

#### 6.3.3. Example 3: The Error-Correcting Code

The classical Ising model is unable to decode an error-correcting code.

A higher-order Ising model [29] has been attempted to decode an error-correcting code. The attempt in [29] incorporated the parity-check matrix into the energy function. Only linear codes have parity-check matrices. No attempts have been reported to decode nonlinear error-correcting codes using a higher-order Ising model.

This paper sets up an energy function to decode a nonlinear error-correcting code, which has a code length of 6 and a code Hamming distance of 3. This code has four codewords and is able to correct for one error in one block. In Table 12, all possible binary strings of length are used for the input; our algorithm takes at most two steps to find the closest codeword. If the input message block contains one error, our algorithm takes one step to find its closest codeword.

Let us consider the most famous linear error-correcting codes—the Hamming H(n,k) codes—where n is the code length in the form of $n=2^{r}-1$ and k is the message length in the form of $k=2^{r}-r-1$, with r being an integer greater than or equal to 2. The code distance is 3 and it is able to correct for one error per block. The total number of codewords is $m=2^{k}$. The size of the parity-check matrix H is r×n. The decode runtime for this linear Hamming code is O(rn)=O(nlogn).

We need to run one step of our proposed algorithm to decode the Hamming H(n,k) codes. The computation runtime for one step (i.e., one iteration) in our proposed algorithm is to evaluate the energy function, which calculates the discrepancies to each codeword. Therefore, the computation runtime for each step is O(mn), which is comparable with O(rn).

In a linear or nonlinear error-correcting code of length n, let the number of codewords be m and we assume that the code is able to correct for t errors. Each step of the proposed algorithm reduces the Hamming distance between the input message and the codeword by 1. Thus, the proposed algorithm takes t steps to correct all t errors. The runtime for this code to correct all t errors is O(tmn). In other words, the proposed decoding algorithm scales well with a polynomial runtime with respect to n,m, and t.

In general, for an n-bit problem with m stable solutions, the upper bound for the monomial count can be calculated by binomial coefficients as

(32) m=C(n−1,0)+C(n−1,1)+⋯+C(n−1,M−1)ifM≤n;

(33) $$m=2^{n-1}\;\text{if}\;M>n.$$

For the OR-gate and XOR-gate examples, n=3,M=4; the monomial count’s upper bound is $2^{n-1}=4$. The actual monomial count for these two examples is 4. For the error-correcting code example, n=6,M=4; the monomial count’s upper bound is C(5,0)+C(5,1)+C(5,2)+C(5,3)=1+5+10+10=26. The actual monomial count is 21.

## 7. Conclusions

The traditional Ising model is defined by a quadratic energy function. The purpose of the Ising model is to find a desired pattern that is closest to the input pattern. An Ising model can perform this task only if the desired patterns are local minima of the quadratic energy function. Generally speaking, this special quadratic energy function does not exist. In other words, the famous Hebbian rule to find the Ising model does not always work.

Compared with the classical Ising model, the most significant advantage of the proposed model is that the extended model can solve many problems (such as XOR-gate problem and decoding problem of error-correcting codes), for which the classical Ising model cannot. The common optimization methods try to find a global solution by using random perturbations. The only known solution is exhaustive search, which is NP-hard. In our practical error-correcting code decoding problem, we formulate the problem in such a way that our local minimum is a global minimum. Therefore, the global minimization problem is converted into a much easier and tractable local minimization problem.

The innovation of this paper is to increase the order of the energy function so that we can find an energy function so that its local minima correspond to the desired patterns. This paper gives an explicit formula to create such an energy function E. In this newly proposed energy function, all local minima are the global minima in the sense that they all have E=0. If for a pattern x with E(x)≠0, then this pattern is not a desired pattern. We have derived explicit closed-form update formulas for the extended Ising model. Our update formulas are not gradient based. We believe that for binary applications, it is more efficient and more effective to select the variable value that makes the energy value smaller by comparing the two possible values, either 0 or 1.

The well-known Boltzmann algorithm is stochastic and non-deterministic. On the other hand, our proposed algorithm is deterministic, and it is guaranteed that the algorithm minimizes the energy function. The Ising model has update formulas in the form of a nonlinear activation function of a linear function of binary variables. The extension of the Ising model allows the update formulas in the form of a nonlinear activation function of a weighted sum of products of the binary variables.

The impact of this research is to qualify more applications for the tasks of finding desired patterns. We do not have restrictions on what the desired patterns are. Those patterns may not be orthogonal or unrelated.

One potential practical application of the proposed model is in error-correcting codes [30,31]. The codewords are the desired patterns and the received messages are used as the input to the model. The model will decode the noise-corrupted messages. Error-correcting code decoding is a perfect practical application for our extended model because the goal of decoding is to find a local minimum of the energy function.

Now, we discuss whether our method is related to the Quadratic Unconstrained Binary Optimization (QUBO) methods. QUBO is a mathematical model used for solving complex optimization problems by expressing them as a quadratic function with binary (0 or 1) variables and no constraints. Binary optimization problems are special cases of discrete optimization problems. The goal of these problems is to find the global minimum (or maximum) solution of an unconstrained quadratic function, which may have multiple local minima. The search for the global minimum is difficult and is, in general, an NP-hard problem, as shown in the references [32–34].

The error-correcting decoding problem as formulated in our paper is not a general QUBO problem. The trick is in a special way of forming the energy function. Our way of forming the energy function guarantees that the energy function is zero of a string if and only if this string is a codeword. Let us consider an error-correcting code that is able to correct for one error and consider a received message block, x, of length n.

If E(x)=0, then x is a codeword.

If E(x)>0, then x is not a codeword. If x has one error in it, it is one Hamming distance away from a codeword $\hat{x}$ with $E(\hat{x})=0$. Each step of the proposed algorithm reduces the Hamming distance by 1. Because the error-correcting code can correct for one error, the code distance must be at least 3. In the distance 1 neighborhood of x, there is only one codeword. Thus, x is correctly decoded as $\hat{x}$.

If E(x)>0, and after one iteration of the algorithm, x becomes $\hat{x}$. At this time, if $E(\hat{x})>0$, then x must contain more than one error and we cannot uniquely decode x.

In this error-correcting code decoding situation, we are only interested in finding the minimum in a small neighborhood centered at x. This small neighborhood consists of n strings. One of the n strings is the codeword, and the other n−1 string are not. This small neighborhood constraint makes our problem different from the Quadratic Unconstrained Binary Optimization (QUBO) problem, which is a difficult mathematical problem. We have reduced the error-correcting code decoding problem from a general QUBO problem to a local problem, which is much easier to solve.

Another potential practical application is pattern recognition, which can be DNA segment recognition, signal denoising, and the like.

## Figures and Tables

**Table 1.** The truth table for the logic OR function in {−1, 1}.

$x_{1}$	$x_{2}$	$x_{3}$
−1	−1	−1
−1	1	1
1	−1	1
1	1	1

**Table 2.** The logic OR function in {−1, 1}. The shading indicates stable solutions.

$x_{1}$	$x_{2}$	$x_{3}$	E
−1	−1	−1	−1.5
−1	1	1	−0.5
1	−1	1	−0.5
1	1	1	−2.5
−1	−1	1	1.5
−1	1	−1	0.5
1	−1	−1	0.5
1	1	−1	2.5

**Table 3.** Update $x_{3}$, while $x_{1}$ and $x_{2}$ are given.

$x_{1}$	$x_{2}$	$x_{1}+x_{2}+0.5$	$sign\left(-\frac{\partial E}{\partial x_{3}}\right)$
−1	−1	−1.5	−1
−1	1	0.5	1
1	−1	0.5	1
1	1	2.5	1

**Table 4.** The truth table for the logic XOR function in {−1, 1}.

$x_{1}$	$x_{2}$	$x_{3}$
−1	−1	−1
−1	1	1
1	−1	1
1	1	−1

**Table 5.** The truth table for the logic OR function in {0, 1}.

$x_{1}$	$x_{2}$	$x_{3}$
0	0	0
0	1	1
1	0	1
1	1	1

**Table 6.** The logic OR function in {0, 1}. The shading indicates stable solutions.

$x_{1}$	$x_{2}$	$x_{3}$	E
0	0	0	0
0	1	1	0
1	0	1	0
1	1	1	0
0	0	1	2
0	1	0	6
1	0	0	6
1	1	0	8

**Table 7.** The truth table for the logic XOR function in {0, 1}.

$x_{1}$	$x_{2}$	$x_{3}$
0	0	0
0	1	1
1	0	1
1	1	0

**Table 8.** The logic XOR function in {0, 1}. The shading indicates stable solutions.

$x_{1}$	$x_{2}$	$x_{3}$	E
0	0	0	0
0	1	1	0
1	0	1	0
1	1	0	0
0	0	1	3
0	1	0	3
1	0	0	3
1	1	1	3

**Table 9.** Codewords.

Index	Codewords
i	$c_{1}^{\left(i\right)}c_{2}^{\left(i\right)}c_{3}^{\left(i\right)}c_{4}^{\left(i\right)}c_{5}^{\left(i\right)}c_{6}^{\left(i\right)}$
1	000000
2	111000
3	000111
4	111111

**Table 10.** The OR gate network update till convergence.

Step 0 $\left(x_{1}x_{2}x_{3}\right)$	Step 1 $\left(x_{1}x_{2}x_{3}\right)$	Step 2 $\left(x_{1}x_{2}x_{3}\right)$
000 (stable)		
001	101 (stable)	
	011 (stable)	
	000 (stable)	
010	000 (stable)	
	011 (stable)	
011 (stable)		
100	000 (stable)	
	101 (stable)	
101 (stable)		
110	010	000 (stable)
		011 (stable)
	100	000 (stable)
		101 (stable)
	111 (stable)	
111 (stable)		

**Table 11.** The XOR gate network update till convergence.

Step 0 ${(x}_{1}x_{2}x_{3})$	Step 1 $\left(x_{1}x_{2}x_{3}\right)$
000 (stable)	
001	101 (stable)
	011 (stable)
	000 (stable)
010	000 (stable)
	011 (stable)
011 (stable)	
100	000 (stable)
	110 (stable)
	101 (stable)
101 (stable)	
110 (stable)	
111	011 (stable)
	101 (stable)
	110 (stable)

**Table 12.** The error-correcting code decoding update till convergence.

Step 0 $x_{1}x_{2}x_{3}x_{4}x_{5}x_{6}$	Step 1 $x_{1}x_{2}x_{3}x_{4}x_{5}x_{6}$	Step 2 $x_{1}x_{2}x_{3}x_{4}x_{5}x_{6}$
000000 (codeword)		
000001 (non-codeword)	000000 (codeword)	
000010 (non-codeword)	000000 (codeword)	
000011 (non-codeword)	000111 (codeword)	
000100 (non-codeword)	000000 (codeword)	
000101 (non-codeword)	000111 (codeword)	
000110 (non-codeword)	000111 (codeword)	
000111 (codeword)		
001000 (non-codeword)	000000 (codeword)	
001001 (non-codeword)	000001 (non-codeword)	000000 (codeword)
001010 (non-codeword)	000010 (non-codeword)	000000 (codeword)
001011 (non-codeword)	000011 (non-codeword)	000111 (codeword)
001100 (non-codeword)	000100 (non-codeword)	000000 (codeword)
001101 (non-codeword)	000101 (non-codeword)	000111 (codeword)
001110 (non-codeword)	000110 (non-codeword)	000111 (codeword)
001111 (non-codeword)	000111 (codeword)	
010000 (non-codeword)	000000 (codeword)	
010001 (non-codeword)	000001 (non-codeword)	000000 (codeword)
010010 (non-codeword)	000010 (non-codeword)	000000 (codeword)
010011 (non-codeword)	000011 (non-codeword)	000111 (codeword)
010100 (non-codeword)	000100 (non-codeword)	000000 (codeword)
010101 (non-codeword)	000101 (non-codeword)	000111 (codeword)
010110 (non-codeword)	000110 (non-codeword)	000111 (codeword)
010111 (non-codeword)	000111 (codeword)	
011000 (non-codeword)	111000 (codeword)	
011001 (non-codeword)	111001 (non-codeword)	111000 (codeword)
011010 (non-codeword)	111010 (non-codeword)	111000 (codeword)
011011 (non-codeword)	111011 (non-codeword)	111111 (codeword)
011100 (non-codeword)	111100 (non-codeword)	111000 (codeword)
011101 (non-codeword)	111101 (non-codeword)	111111 (codeword)
011110 (non-codeword)	111110 (non-codeword)	111111 (codeword)
011111 (non-codeword)	111111 (codeword)	
100000 (non-codeword)	000000 (codeword)	
100001 (non-codeword)	000001 (non-codeword)	000000 (codeword)
100010 (non-codeword)	000010 (non-codeword)	000000 (codeword)
100011 (non-codeword)	000011 (non-codeword)	000111 (codeword)
100100 (non-codeword)	000100 (non-codeword)	000000 (codeword)
100101 (non-codeword)	000101 (non-codeword)	000111 (codeword)
100110 (non-codeword)	000110 (non-codeword)	000111 (codeword)
100111 (non-codeword)	000111 (codeword)	
101000 (non-codeword)	111000 (codeword)	
101001 (non-codeword)	111001 (non-codeword)	111000 (codeword)
101010 (non-codeword)	111010 (non-codeword)	111000 (codeword)
101011 (non-codeword)	111011 (non-codeword)	111111 (codeword)
101100 (non-codeword)	111100 (non-codeword)	111000 (codeword)
101101 (non-codeword)	111101 (non-codeword)	111111 (codeword)
101110 (non-codeword)	111110 (non-codeword)	111111 (codeword)
101111 (non-codeword)	111111 (codeword)	
110000 (non-codeword)	111000 (codeword)	
110001 (non-codeword)	111001 (non-codeword)	111000 (codeword)
110010 (non-codeword)	111010 (non-codeword)	111000 (codeword)
110011 (non-codeword)	111011 (non-codeword)	111111 (codeword)
110100 (non-codeword)	111100 (non-codeword)	111000 (codeword)
110101 (non-codeword)	111101 (non-codeword)	111111 (codeword)
110110 (non-codeword)	111110 (non-codeword)	111111 (codeword)
110111 (non-codeword)	111111 (codeword)	
111000 (codeword)		
111001 (non-codeword)	111000 (codeword)	
111010 (non-codeword)	111000 (codeword)	
111011 (non-codeword)	111111 (codeword)	
111100 (non-codeword)	111000 (codeword)	
111101 (non-codeword)	111111 (codeword)	
111110 (non-codeword)	111111 (codeword)	
111111 (codeword)		

## Data Availability

No new data were created or analyzed in this study. Data sharing is not applicable to this article.

## Appendix A. Closed-Form Formula for the Weights

In Section 2, we had two equivalent expressions for the new energy function E(x):

$$E\left(x\right)=\prod_{i=1}^{M}{\left\|x-x^{S_{i}}\right\|}^{2}=\prod_{i=1}^{M}\sum_{j=1}^{n}{\left(x_{j}-x_{j}^{S_{i}}\right)}^{2},$$

And

$$E\left(x\right)=\sum_{k=0}^{2^{n}-1}a_{k}\prod_{i=1}^{n}x_{i}^{k_{i}}=\sum_{k=0}^{2^{n}-1}a_{k}x_{1}^{k_{1}}x_{2}^{k_{2}}\ldots x_{n}^{k_{n}}.$$

By comparing (11) and (13), we can obtain a closed-form formula to explicitly calculate the coefficients $a_{k}$ in (13). In the matrix form, (13) is equivalent to

(A1)
BA=E,

where

(A2)
$$A={\left[a_{0},a_{1},\ldots ,a_{2^{n}-1}\right]}^{T},$$

(A3)
$$E={\left[E(0),E(1),\ldots ,E\left(2^{n}-1\right)\right]}^{T},$$

(A4)
$$b_{ij}=\prod_{m=1}^{n}i_{m}^{j_{m}}.$$

In (A1), A is a vector, E is a vector, and B is a matrix. In (A4), $b_{ij}$ is a general element in the matrix B, the integer i is expressed in n-bit binary $i_{1}i_{2}\ldots i_{2^{n}-1}$, and the integer j is expressed in n-bit binary $j_{1}j_{2}\ldots j_{2^{n}-1}$. Short-hand notation is used in (A3). For n=3,E(0) represents E(0,0,0);E(1) represents E(0,0,1);E(2) represents E(0,1,0);E(3) represents E(0,1,1);E(4) represents E(1,0,0);E(5) represents E(1,0,1);E(6) represents E(1,1,0);E(7) represents E(1,1,1). Two examples of calculating $b_{ij}$ are

(A5)
$$b_{0,2}=b_{000,010}=0^{0}\times 0^{1}\times 0^{0}=1\times 0\times 1=0;$$

(A6)
$$b_{1,3}=b_{001,011}=0^{0}\times 0^{1}\times 1^{1}=1\times 0\times 1=0.$$

Due to the definition (A4), matrix B can be constructed recursively as

(A7)
$$B_{0}=[1],$$

(A8)
$$\begin{array}{c} B_{2^{n}}=\left[\begin{array}{ll} B_{2^{n-1}} & 0_{2^{n-1}}\\ B_{2^{n-1}} & B_{2^{n-1}} \end{array}\right], \end{array}$$

where $B_{2^{n}}$ is the matrix B for n-bit strings; the size of B is $2^{n}\times 2^{n};0_{2^{n-1}}$ is a $2^{n-1}\times 2^{n-1}$ zero matrix. Matrix B is a lower-triangle matrix with all-one diagonal elements.

The new explicit formula for the weights $a_{k}$ is

(A9)
$$A=B^{-1}E.$$

After the coefficients, $a_{k}$’s, are determined, the update formula is derived from (16) as

(A10)
$$x_{m}=\text{step}\left(E_{m}^{0}-E_{m}^{1}\right)\;=\text{step}\left({\left.\sum_{k=0}^{2^{n}-1}a_{k}\prod_{i=1}^{n}x_{i}^{k_{i}}\right|}_{x_{m}=0}-{\left.\sum_{k=0}^{2^{n}-1}a_{k}\prod_{i=1}^{n}x_{i}^{k_{i}}\right|}_{x_{m}=1}\right)$$

Notice that

(A11)
$$0^{k_{m}}-1^{k_{m}}=-k_{m},\;\text{with}\;k_{m}\in \{0,1\}.$$

Then the update formula (A10) becomes

(A12)
$$x_{m}=\text{step}\left(-\sum_{k=0}^{2^{n}-1}k_{m}a_{k}\prod_{\begin{array}{c} i=1\\ i\neq m \end{array}}^{n}x_{i}^{k_{i}}\right).$$

Formulas (A9) and (A12) are the main results of this paper. We now apply (A9) and (A12) to our two examples. In fact, the sum in (A12) can be easily obtained from (13) by discarding all terms without $x_{m}$ and by changing the sign of the sum.

### A.1. Example 1: A logic OR gate

First, we form matrix B by using all eight possible 3-bit inputs.

In Table A1, the first row is labeled as binary numbers in the ascending order: 000, 001, …, 111 from left to right. These binary numbers indicate the variables in the second row. For example, 001 indicates $x_{3}^{0}x_{2}^{0}x_{1}^{1}=x_{1}$, and 110 indicates $x_{3}^{1}x_{2}^{1}x_{1}^{0}=x_{3}x_{2}$, and so on.

The first (left) eight columns of Table A1 construct the matrix B:

(A13)
$$B=\left[\begin{array}{llllllll} 1 & 0 & 0 & 0 & 0 & 0 & 0 & 0\\ 1 & 1 & 0 & 0 & 0 & 0 & 0 & 0\\ 1 & 0 & 1 & 0 & 0 & 0 & 0 & 0\\ 1 & 1 & 1 & 1 & 0 & 0 & 0 & 0\\ 1 & 0 & 0 & 0 & 1 & 0 & 0 & 0\\ 1 & 1 & 0 & 0 & 1 & 1 & 0 & 0\\ 1 & 0 & 1 & 0 & 1 & 0 & 1 & 0\\ 1 & 1 & 1 & 1 & 1 & 1 & 1 & 1 \end{array}\right].$$

The last (right) column of Table A1 constructs the vector E:

(A14)
$$E=\left[\begin{array}{l} 0\\ 6\\ 6\\ 8\\ 2\\ 0\\ 0\\ 0 \end{array}\right]$$

Thus, according to (A9), we have

(A15)
$$A=B^{-1}E=\left[\begin{array}{c} 0\\ 6\\ 6\\ -4\\ 2\\ -8\\ -8\\ 6 \end{array}\right]$$

The coefficients given in (A15) directly produce the energy function according to (13)

(A16)
$$E(x)=6x_{1}+6x_{2}-4x_{1}x_{2}+2x_{3}-8x_{1}x_{3}-8x_{2}x_{3}+6x_{1}x_{2}x_{3},$$

which is the same as (14). The update formulas are obtained according to (A12):

(A17)
$$x_{1}=\text{step}\left(-6+4x_{2}+8x_{3}-6x_{2}x_{3}\right);$$

(A18)
$$x_{2}=\text{step}\left(-6+4x_{1}+8x_{3}-6x_{1}x_{3}\right);$$

(A19)
$$x_{3}=\text{step}\left(-2+8x_{1}+8x_{2}-6x_{1}x_{2}\right).$$

The update formulas (A17), (A18), and (A19) are the same as (18), (19), and (20), respectively. In fact, (A17) is first obtained from (A16) by discarding all terms without $x_{1}$, that is,

(A20)
$${\text{temp}}_{1,1}=6x_{1}-4x_{1}x_{2}-8x_{1}x_{3}+6x_{1}x_{2}x_{3}.$$

Then replace $x_{1}$ by −1,

(A21)
$${\text{temp}}_{1,2}=-6+4x_{2}+8x_{3}-6x_{2}x_{3}.$$

The update formula for $x_{1}$ is

(A22)
$$x_{1}=\text{step}\left({\text{temp}}_{1,2}\right).$$

Similarly, (A18) is first obtained from (A12) by discarding all terms without $x_{2}$, that is,

(A23)
$${\text{temp}}_{2,1}=6x_{2}-4x_{1}x_{2}-8x_{2}x_{3}+6x_{1}x_{2}x_{3}.$$

Then replace $x_{2}$ by −1,

(A24)
$${\text{temp}}_{2,2}=-6+4x_{1}+8x_{3}-6x_{1}x_{3}.$$

The update formula for $x_{2}$ is

(A25)
$$x_{2}=\text{step}\left({\text{temp}}_{2,2}\right).$$

Again, (A19) is first obtained from (A12) by discarding all terms without $x_{3}$, that is,

(A26)
$${\text{temp}}_{3,1}=2x_{3}-8x_{1}x_{3}-8x_{2}x_{3}+6x_{1}x_{2}x_{3}.$$

Then replace $x_{3}$ by −1,

(A27)
$${\text{temp}}_{3,2}=-2+8x_{1}+8x_{2}-6x_{1}x_{2}.$$

The update formula for $x_{3}$ is

(A28)
$$x_{3}=\text{step}\left({\text{temp}}_{3,2}\right).$$

**Table A1.** The logic OR function in {0, 1}.

000	001	010	011	100	101	110	111	
1	$x_{1}$	$x_{2}$	$x_{2}x_{1}$	$x_{3}$	$x_{3}x_{1}$	$x_{3}x_{2}$	$x_{3}x_{2}x_{1}$	E
1	0	0	0	0	0	0	0	0
1	1	0	0	0	0	0	0	6
1	0	1	0	0	0	0	0	6
1	1	1	1	0	0	0	0	8
1	0	0	0	1	0	0	0	2
1	1	0	0	1	1	0	0	0
1	0	1	0	1	0	1	0	0
1	1	1	1	1	1	1	1	0

### A.2. Example 2: A logic XOR gate

First, we form matrix B by using all eight possible 3-bit inputs.

The first (left) eight columns of Table A2 construct the matrix B, which is the same as (A13).

The last (right) column of Table A2 constructs the matrix E:

(A29)
$$E=\left[\begin{array}{l} 0\\ 3\\ 3\\ 0\\ 3\\ 0\\ 0\\ 3 \end{array}\right].$$

Thus, according to (A9), we have

(A30)
$$A=B^{-1}E=\left[\begin{array}{c} 0\\ 3\\ 3\\ -6\\ 3\\ -6\\ -6\\ 12 \end{array}\right].$$

The coefficients given in (A30) directly produce the energy function according to (13):

(A31)
$$E(x)=3x_{1}+3x_{2}-6x_{1}x_{2}+3x_{3}-6x_{1}x_{3}-6x_{2}x_{3}+12x_{1}x_{2}x_{3},$$

which is the same as (24). The update formulas are obtained according to (A21):

(A32)
$$x_{1}=\text{step}\left(-3+6x_{2}+6x_{3}-12x_{2}x_{3}\right);$$

(A33)
$$x_{2}=\text{step}\left(-3+6x_{1}+6x_{3}-12x_{1}x_{3}\right);$$

(A34)
$$x_{3}=\text{step}\left(-3+6x_{1}+6x_{2}-12x_{1}x_{2}\right).$$

The update formulas (A32), (A33), and (A34) are the same as (21), (22), and (23), respectively. In fact, (A32) is first obtained from (A31) by discarding all terms without $x_{1}$, that is,

(A35)
$${\text{temp}}_{1,1}=3x_{1}-6x_{1}x_{2}-6x_{1}x_{3}+12x_{1}x_{2}x_{3}.$$

Then replace $x_{1}$ by −1,

(A36)
$${\text{temp}}_{1,2}=-3+6x_{2}+6x_{3}-12x_{2}x_{3}.$$

The update formula for $x_{1}$ is

(A37)
$$x_{1}=\text{step}\left({\text{temp}}_{1,2}\right).$$

Similarly, (A33) is first from (A31) by discarding all terms without $x_{2}$, that is,

(A38)
$${\text{temp}}_{2,1}=3x_{2}-6x_{1}x_{2}-6x_{2}x_{3}+12x_{1}x_{2}x_{3}.$$

Then replace $x_{2}$ by −1,

(A39)
$${\text{temp}}_{2,2}=-3+6x_{1}+6x_{3}-12x_{1}x_{3}.$$

The update formula for $x_{2}$ is

(A40)
$$x_{2}=\text{step}\left({\text{temp}}_{2,2}\right).$$

Again, (A34) is first from (A31) by discarding all terms without $x_{3}$, that is,

(A41)
$${\text{temp}}_{3,1}=3x_{3}-6x_{1}x_{3}-6x_{2}x_{3}+12x_{1}x_{2}x_{3}.$$

Then replace $x_{3}$ by −1,

(A42)
$${\text{temp}}_{3,2}=-3+6x_{1}+6x_{2}-12x_{1}x_{2}.$$

The update formula for $x_{3}$ is

(A43)
$$x_{3}=\text{step}\left({\text{temp}}_{3,2}\right).$$

**Table A2.** The logic XOR function in {0, 1}.

000	001	010	011	100	101	110	111	
1	$x_{1}$	$x_{2}$	$x_{2}x_{1}$	$x_{3}$	$x_{3}x_{1}$	$x_{3}x_{2}$	$x_{3}x_{2}x_{1}$	E
1	0	0	0	0	0	0	0	0
1	1	0	0	0	0	0	0	3
1	0	1	0	0	0	0	0	3
1	1	1	1	0	0	0	0	0
1	0	0	0	1	0	0	0	3
1	1	0	0	1	1	0	0	0
1	0	1	0	1	0	1	0	0
1	1	1	1	1	1	1	1	3

## Abbreviations

The following abbreviations are used in this manuscript:

FPGA: Field Programmable Gate Array
QUBO: Quadratic Unconstrained Binary Optimization
XOR: Exclusive-OR
